# Prognostic Impact of Pelvic Lymph Node Count in Surgically Staged Endometrial Cancer

**DOI:** 10.3390/medicina62020399

**Published:** 2026-02-19

**Authors:** Yakup Yalcin, Kemal Ozerkan

**Affiliations:** Department of Obstetrics and Gynecology, School of Medicine, Bursa Uludag University, Bursa 16110, Turkey

**Keywords:** endometrial cancer, pelvic lymphadenectomy, lymph node count, recurrence, survival

## Abstract

*Background and Objectives:* The prognostic significance of pelvic lymph node (PLN) count in surgically staged endometrial cancer remains controversial. This study aimed to evaluate the impact of PLN count on overall survival (OS), disease-free survival (DFS), and recurrence patterns in a large cohort of patients with endometrial cancer. *Materials and Methods:* This retrospective cohort study included 560 patients with endometrial cancer who underwent total hysterectomy, bilateral salpingo-oophorectomy, and pelvic and/or para-aortic lymph node assessment between January 2005 and May 2025 at a tertiary referral center. Patients were stratified according to the number of harvested pelvic lymph nodes (≤20 vs. >20). Clinicopathological characteristics, adjuvant treatments, recurrence patterns, and survival outcomes were analyzed. Survival analyses were performed using Kaplan–Meier estimates and Cox proportional hazards regression models. *Results:* Of the 560 patients, 262 (46.8%) had ≤20 pelvic lymph nodes harvested and 298 (53.2%) had >20. The median follow-up duration was 64.5 months. Patients with >20 pelvic lymph nodes had larger tumors, higher FIGO stage, and more frequent para-aortic lymphadenectomy. In multivariate analysis, age, non-endometrioid histology, advanced FIGO stage, tumor grade, and lymphatic metastasis were independently associated with both OS and DFS. Pelvic lymph node count was not independently associated with OS or DFS. Overall recurrence rates were similar between groups; however, recurrence patterns differed significantly, with distant recurrences more frequent in the ≤20 PLN group and local recurrences more common in the >20 PLN group. *Conclusions:* In surgically staged endometrial cancer, a higher pelvic lymph node count (>20 nodes) was not independently associated with survival or recurrence outcomes after adjustment for established prognostic factors, although recurrence patterns differed between groups. Survival was primarily determined by age, histologic subtype, FIGO stage, tumor grade, and lymphatic metastasis. Pelvic lymph node count appears to reflect surgical staging intensity and intraoperative risk assessment rather than serving as an independent determinant of prognosis.

## 1. Introduction

Endometrial cancer is the most common gynecologic malignancy in developed countries, and its incidence continues to increase worldwide due to the aging population and rising obesity rates [1]. Surgical staging remains the cornerstone of management and traditionally includes total hysterectomy, bilateral salpingo-oophorectomy, and assessment of lymph node status, which is one of the most important prognostic factors in endometrial cancer [2,3,4].

The role of lymphadenectomy in endometrial cancer, however, has long been a subject of debate. While lymph node assessment provides critical staging information and guides adjuvant treatment decisions, systematic lymphadenectomy is associated with increased operative time, blood loss, and postoperative morbidity, including lymphedema and lymphocyst formation [5,6,7]. Obesity, increased uterine size, and reduced pelvic working space can significantly increase surgical complexity, even in experienced hands, and contribute to variability in the number of lymph nodes collected, limiting the comprehensiveness of lymph node dissection. Accordingly, the number of pelvic lymph nodes should be considered not only as a reflection of the surgical aim but also as an indicator influenced by individual anatomical and technical factors [8]. Two landmark randomized trials failed to demonstrate a survival benefit for routine pelvic lymphadenectomy, particularly in early-stage disease, leading to ongoing controversy regarding its therapeutic value [9,10].

In recent years, sentinel lymph node (SLN) mapping has emerged as an alternative staging strategy, offering accurate nodal assessment with significantly reduced surgical morbidity [11]. SLN mapping is now widely accepted for low-risk endometrial cancer and increasingly incorporated into international guidelines [12,13]. Nevertheless, uncertainty persists regarding optimal nodal assessment in patients with intermediate- and high-risk disease, where systematic lymphadenectomy is still frequently performed in clinical practice [14,15].

Beyond the binary question of whether lymphadenectomy should be performed, increasing attention has been directed toward the extent and thoroughness of lymph node dissection. The prognostic relevance of pelvic lymph node count in endometrial cancer remains controversial. Several retrospective and population-based studies have suggested that a higher number of harvested lymph nodes may be associated with improved survival, even in patients with pathologically node-negative disease [16,17,18]. Proposed explanations include improved detection of occult metastases, more accurate staging (stage migration), and a potential therapeutic effect through removal of microscopic disease. However, these findings remain inconsistent, and the clinical relevance of lymph node count as an independent prognostic factor is not fully established [19,20]. Previous studies have reported heterogeneous cut-off values, ranging from 10 to more than 25–30 pelvic lymph nodes, reflecting differences in patient characteristics, surgical practice, and study design [16,17,18,19,20].

Given the persistent controversy surrounding the prognostic and therapeutic implications of lymphadenectomy, the present study aimed to evaluate the impact of pelvic lymph node (PLN) count on overall survival (OS) and disease-free survival (DFS) in a large cohort of patients with surgically staged endometrial cancer. By comparing outcomes between patients stratified according to pelvic lymph node count, we sought to evaluate whether the extent of pelvic lymph node dissection influences survival outcomes and recurrence patterns in contemporary clinical practice.

## 2. Materials and Methods

### 2.1. Study Design and Patient Population

This retrospective cohort study evaluated patients diagnosed with endometrial cancer who underwent primary surgical treatment between January 2005 and May 2025 at a tertiary referral center. Patient recruitment was retrospective and consecutive. All eligible patients who underwent primary surgical treatment for endometrial cancer during the study period were screened for inclusion to minimize selection bias.

Inclusion criteria were as follows: (i) histologically confirmed endometrial carcinoma; (ii) primary surgical treatment consisting of total hysterectomy and bilateral salpingo-oophorectomy; (iii) pelvic lymph node assessment with or without paraaortic lymphadenectomy performed as part of surgical staging; and (iv) availability of sufficient complete clinicopathological data and follow-up information for survival analysis.

Exclusion criteria included: (i) absence of pelvic lymph node assessment; (ii) incomplete or missing pathological data regarding lymph node status or number of lymph nodes collected; (iii) insufficient follow-up time or missing survival outcome data; (iv) prior history of pelvic or paraaortic radiotherapy or neoadjuvant treatment that could alter lymph node yield; and (v) non-primary surgery or palliative surgery. Patients with concomitant malignancies or recurrent endometrial cancer at the time of referral were also excluded from the analysis. 

The study was conducted at a high-volume tertiary referral center where surgical treatment of endometrial cancer is routinely performed. The average annual number of endometrial cancer cases is around 50. All procedures were performed by 2 or 3 specialist gynecological oncologists with significant experience in gynecological oncological surgery. Throughout the study period, surgeries were performed by surgeons with an average of over 10 years of professional experience in the field of gynecological oncology. Surgical techniques and pathological evaluation were performed in accordance with standardized institutional protocols, and consistency in operative and histopathological evaluation was maintained throughout the study period.

Para-aortic lymphadenectomy was performed selectively based on the patient’s individual risk profile and the surgeon’s intraoperative clinical judgment. The primary reasons for omitting para-aortic lymphadenectomy were intraoperative and patient-related factors that were considered to increase surgical risk or perioperative morbidity. In particular, para-aortic dissection was avoided in patients who were unlikely to tolerate prolonged operative time, those with significant comorbidities, or patients with a high body mass index (BMI) that could increase surgical complexity and the risk of postoperative complications. Patients with incomplete clinical data or missing lymph node information were excluded from the analysis. For survival analyses, patients were stratified according to the number of pelvic lymph nodes harvested into two groups: ≤20 pelvic lymph nodes and >20 pelvic lymph nodes. Given the lack of a universally accepted biological threshold and the heterogeneity in the literature, a cut-off of 20 harvested pelvic lymph nodes was predefined for stratification. This threshold has been frequently used in widely cited studies as a pragmatic indicator of extensive pelvic lymphadenectomy rather than a biologically determined prognostic value, allowing comparability with prior analyses [17,18,21]. To further address concerns regarding potential cut-off dependency, an exploratory sensitivity analysis using alternative pelvic lymph node thresholds was conducted and is detailed in the Statistical Analysis section.

### 2.2. Data Collection

Clinicopathological variables were retrieved from institutional medical records and pathology reports, including age at diagnosis, body mass index (BMI), preoperative Ca 125 levels, tumor size, FIGO stage (2009 classification), histologic subtype, tumor grade, depth of myometrial invasion, lymphovascular space invasion (LVSI), cervical stromal invasion, para-aortic lymphadenectomy status, number of pelvic and para-aortic lymph nodes harvested, lymphatic metastasis patterns, surgical approach, adjuvant treatment modalities, recurrence patterns, and survival outcomes.

Overall survival was defined as the time from primary surgery to death from any cause or last follow-up. Disease-free survival was defined as the time from primary surgery to the first documented recurrence or last follow-up without evidence of disease.

### 2.3. Statistical Analysis

All statistical analyses were conducted using IBM SPSS Statistics for Windows, version 26.0 (IBM Corp., Armonk, NY, USA). Continuous variables were summarized as medians with ranges and compared using the Mann–Whitney U test. Categorical variables were presented as frequencies and percentages and compared using the chi-squared test or Fisher’s exact test, as appropriate. Survival curves for OS and DFS were estimated using the Kaplan–Meier method and compared between groups using the log-rank test. Factors associated with OS and DFS were evaluated using univariate and multivariate Cox proportional hazards regression models, with results reported as hazard ratios (HRs) and 95% confidence intervals (CIs). Variables with clinical relevance or statistical significance in univariate analyses were entered into multivariate models. A two-sided *p* value of <0.05 was considered statistically significant.

### 2.4. Sensitivity Analysis of Pelvic Lymph Node Cut-Offs

To evaluate whether the study findings were dependent on the predefined pelvic lymph node cut-off, an exploratory sensitivity analysis was performed. Pelvic lymph node counts were iteratively dichotomized across a broad range of alternative thresholds, including commonly used cut-offs reported in the literature (e.g., 15 and 25 nodes), as well as intermediate values. For each threshold, survival-related differences between groups were assessed. These analyses were conducted for exploratory purposes and are presented in the Supplementary Materials to avoid data-driven overinterpretation.

Variables included in the multivariate Cox proportional hazards regression models were selected based on a combination of clinical relevance and univariate analysis results. Factors known to be associated with survival outcomes in endometrial cancer, such as age, histologic subtype, FIGO stage, tumor grade, lymphatic metastasis, and adjuvant treatment, were entered into the multivariate models regardless of their statistical significance in univariate analysis. This approach was chosen to adequately control for potential confounding and to ensure adjustment for clinically important variables. Accordingly, adjuvant treatment modalities were retained in the final models given their established impact on survival outcomes and their close association with disease stage and risk stratification, even when not independently significant after adjustment. The proportional hazards assumption was assessed using log-minus-log survival plots. Analyses were conducted using a complete-case approach, as missing data were minimal.

### 2.5. Ethical Approval

This study was approved by the local Ethics Committee (Ethics Committee Approval No: 2025/802/14-21). Due to the retrospective nature of the study, the requirement for informed consent was waived.

## 3. Results

### 3.1. Patient Characteristics

A total of 560 patients were included in the analysis, of whom 262 (46.8%) had ≤20 pelvic lymph nodes harvested and 298 (53.2%) had >20 pelvic lymph nodes harvested. The median follow-up duration for the entire cohort was 64.5 months. The median age was identical between the two groups (63.0 years in both groups; *p* = 0.323). Similarly, median body mass index did not differ significantly (34.3 vs. 34.0 kg/m^2^; *p* = 0.764). Median preoperative CA-125 levels were numerically higher in the >20 pelvic LN group but did not reach statistical significance (17.3 vs. 19.5 U/mL; *p* = 0.078). Patients with >20 pelvic lymph nodes had a significantly larger median tumor size compared with those with ≤20 nodes (4.2 vs. 3.7 cm; *p* = 0.002). The distribution of FIGO stage differed significantly between groups (*p* < 0.001), with a higher proportion of stage III disease observed in the >20 LN group (22.5% vs. 13.7%).

Histologic subtype and FIGO grade distributions were comparable between groups (both *p* > 0.45). Although deep myometrial invasion (>50%) tended to be more frequent in the >20 LN group (35.2% vs. 27.9%), this difference did not reach statistical significance (*p* = 0.075). Rates of lymphovascular space invasion and cervical stromal invasion were similar between groups. Para-aortic lymphadenectomy was performed significantly more often in patients with >20 pelvic lymph nodes (84.9% vs. 70.1%; *p* < 0.001), and the median number of harvested para-aortic lymph nodes was higher in this group (18 vs. 11; *p* < 0.001). Lymphatic metastasis patterns differed significantly (*p* = 0.006), with combined pelvic and para-aortic metastases being more frequent in the >20 LN group (9.4% vs. 3.1%). Adjuvant treatment strategies differed between groups (*p* = 0.048), with combined chemotherapy and radiotherapy more frequently administered in patients with >20 pelvic lymph nodes (Table 1).

### 3.2. Overall Survival

In univariate analysis, increasing age, higher Ca 125 levels, larger tumor size, non-endometrioid histology, lymphatic metastasis, advanced FIGO stage, deep myometrial invasion, presence of LVSI, grade 3 disease, and combined chemoradiotherapy were significantly associated with worse overall survival (*p* < 0.05). In the multivariate Cox regression model, age (HR 1.078 per year, *p* < 0.001), Ca 125 (HR 1.003 per unit, *p* = 0.034), non-endometrioid histology (HR 2.343, *p* < 0.001), pelvic and para-aortic lymphatic metastasis (HR 3.745, *p* < 0.001), FIGO stage III (HR 2.213, *p* < 0.001), grade 3 disease (HR 2.845, *p* < 0.001), and receipt of combined chemotherapy and radiotherapy (HR 0.724, *p* = 0.039) remained independently associated with overall survival. The number of harvested pelvic lymph nodes (>20 vs. ≤20), para-aortic lymphadenectomy, and the number of para-aortic lymph nodes removed were not independently associated with overall survival (Table 2).

### 3.3. Disease-Free Survival

Univariate analysis demonstrated that age, tumor size, non-endometrioid histology, lymphatic metastasis, advanced FIGO stage, deep myometrial invasion, LVSI, grade 3 disease, and adjuvant treatment were significantly associated with disease-free survival (*p* < 0.05). In the multivariate model, age (HR 1.032 per year, *p* = 0.034), non-endometrioid histology (HR 3.906, *p* < 0.001), pelvic and para-aortic lymphatic metastasis (HR 5.567, *p* < 0.001), and FIGO stage III (HR 2.860, *p* < 0.001) remained independent predictors of disease-free survival. Tumor size, depth of myometrial invasion, LVSI, grade, and adjuvant treatment were not independently associated with DFS after adjustment. Pelvic lymph node count, para-aortic lymphadenectomy, and number of harvested para-aortic lymph nodes were not independently associated with disease-free survival (Table 3).

### 3.4. Recurrence Patterns

Overall recurrence rates were similar between patients with ≤20 and >20 pelvic lymph nodes (12.2% vs. 11.7%; *p* = 0.968). However, recurrence patterns differed significantly between groups (*p* = 0.019). Patients with ≤20 pelvic lymph nodes experienced a higher proportion of distant recurrences (65.6%), whereas local recurrences were more frequent in patients with >20 pelvic lymph nodes (51.4%). Combined local and distant recurrences were uncommon in both groups (Table 4).

### 3.5. Survival Outcomes According to Pelvic Lymph Node Count

**Figure 1:**
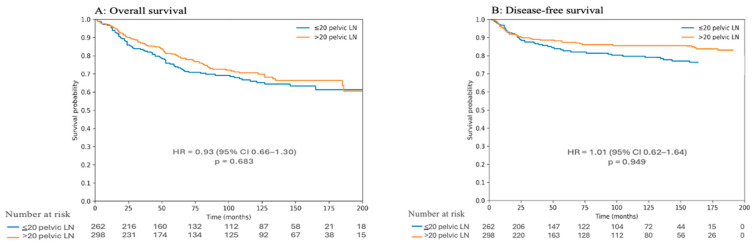
Kaplan–Meier curves comparing overall survival (**A**) and disease-free survival (**B**) according to the number of pelvic lymph nodes harvested (≤20 vs. >20).

Kaplan–Meier survival analyses were performed to compare overall survival (OS) and disease-free survival (DFS) between patients with ≤20 and >20 pelvic lymph nodes harvested (Figure 1). As shown in Figure 1A, there was no statistically significant difference in overall survival between the two groups. Patients with >20 pelvic lymph nodes demonstrated a numerically higher OS compared with those with ≤20 nodes; however, this difference did not reach statistical significance (HR 0.93, 95%CI: 0.66–1.30, *p* = 0.683). Similarly, disease-free survival did not differ significantly between groups (Figure 1B). Although patients with >20 pelvic lymph nodes harvested tended to have higher DFS over time, the difference compared with the ≤20 pelvic lymph node group was not statistically significant (HR 1.01, 95%CI: 0.62–1.64, *p* = 0.949). Overall, these findings indicate that a higher pelvic lymph node count (>20 nodes) was not associated with a statistically significant improvement in OS or DFS, despite a trend toward more favorable survival outcomes.

Exploratory sensitivity analyses demonstrated that the magnitude of survival-related differences varied across pelvic lymph node thresholds. The most pronounced statistical separation was observed around the predefined cut-off of 20 pelvic lymph nodes, whereas adjacent and more distant thresholds demonstrated weaker or inconsistent associations (Supplementary Table S1 and Figure S1).

## 4. Discussion

In this large retrospective cohort of surgically staged endometrial cancer patients, we evaluated whether PLN count has independent prognostic significance for OS and DFS. The principal finding of our study is that harvesting more than 20 pelvic lymph nodes was not independently associated with improved OS or DFS, despite being correlated with more advanced disease characteristics and more extensive surgical staging. These findings contribute meaningful evidence to the ongoing debate regarding the oncologic significance of extensive lymphadenectomy in endometrial cancer and help to clarify its role within contemporary treatment paradigms.

Chan et al. showed that removing 20 or more lymph nodes improved prognosis in their study of 12,333 patients with endometrial cancer [18]. An analysis using the US Surveillance, Epidemiology and Outcomes (SEER) database found that removing up to 20 pelvic lymph nodes detected positive lymph nodes in 85% of cases. The rate of detection of metastatic lymph nodes was significantly higher in the 20–25 lymph node group compared to the 1–5 lymph node group. They stated that removing 20 or more lymph nodes was sufficient to demonstrate true lymph node negativity [21]. Abu-Rustum et al. reported that, in addition to well-known clinicopathological risk factors for survival, removal of 10 or more regional lymph nodes was associated with improved overall survival in low-stage, elderly patients who did not receive adjuvant therapy or who received brachytherapy alone [22].

In a retrospective analysis, Lutman et al. reported that in stage I and II patients without metastases in the removed lymph nodes, the number of removed lymph nodes was not significantly associated with better prognosis in EC stages 1 and 2. However, they stated that in high-risk histology, the number of removed lymph nodes was an independent prognostic factor for OS and PFS. The study indicated two reasons for the prognostic significance of removing more lymph nodes: firstly, the therapeutic benefit of removing occult lymph node metastasis, and secondly, the ‘stage shift’ effect where removing fewer lymph nodes could lead to missing metastases and patients being classified as lower stage [20]. Another study in patients with surgically staged endometrial cancer showed that lymph node count was not effective in accurately predicting the risk of lymph node metastasis. However, the number of lymph node stations sampled has been shown to be more reliable in predicting lymph node metastases than the number of lymph nodes removed [23]. In our study, our multivariate analyses demonstrated that PLN count (>20 vs. ≤20) did not independently influence OS or DFS after adjustment for established prognostic factors such as age, histology, FIGO stage, grade, and lymphatic metastasis. This finding suggests that the apparent survival advantage reported in some earlier studies may, at least in part, be explained by confounding factors including stage migration and patient selection, rather than a true therapeutic effect of extensive lymph node dissection. Importantly, patients in the >20 LN group in our cohort had larger tumors, a higher proportion of stage III disease, more frequent para-aortic lymphadenectomy, and higher rates of combined pelvic and para-aortic metastases.

Our findings are concordant with the results of the two landmark randomized trials—the Italian trial by Benedetti Panici et al. and the MRC ASTEC trial—which failed to demonstrate a survival benefit for systematic pelvic lymphadenectomy in early-stage endometrial cancer [9,10]. While these trials were criticized for limited para-aortic dissection and inclusion of predominantly low-risk patients, their core message—that routine extensive lymphadenectomy does not improve survival—remains highly relevant. More recent international guidelines, including the ESGO-ESTRO-ESP recommendations, emphasize risk-adapted nodal assessment and increasingly favor SLN mapping over systematic lymphadenectomy, particularly in low- and intermediate-risk disease [4,13]. Our data reinforce this paradigm by demonstrating that even when a high pelvic LN count is achieved, survival outcomes are not independently improved.

In both OS and DFS analyses, lymphatic metastasis emerged as one of the strongest independent prognostic factors, with patients harboring combined pelvic and para-aortic metastases experiencing markedly worse outcomes. This finding is consistent with the established literature and highlights that the prognostic value of nodal assessment lies primarily in accurate detection of metastasis, rather than in the absolute number of nodes removed [2,19]. Another noteworthy finding is that adjuvant treatment patterns differed significantly between groups, with combined chemotherapy and radiotherapy more frequently administered to patients with >20 pelvic LNs. Despite this, pelvic LN count remained non-significant in multivariate survival models. This suggests that appropriate adjuvant therapy, guided by accurate staging and tumor biology, may mitigate any theoretical survival advantage of more extensive nodal dissection. Accordingly, these results support a treatment paradigm that emphasizes individualized adjuvant management guided by accurate staging, rather than routine escalation of surgical extent.

In the study by Konno et al., the authors investigated the correlation between the number of resected pelvic lymph nodes and patterns of recurrence. Their analysis demonstrated no significant association between count of pelvic lymph node resection and recurrence patterns, indicating that the extent of pelvic lymph node dissection did not influence the distribution of recurrence sites in patients with endometrial cancer [17]. In our study, although overall recurrence rates were similar between groups, differences in recurrence patterns were observed. Patients with ≤20 pelvic lymph nodes tended to experience a higher proportion of distant recurrences, whereas local recurrences were more frequent in patients with >20 pelvic lymph nodes. However, these findings should be interpreted with caution, as the number of recurrence events was limited and the analysis was not adjusted for potential confounders, including adjuvant treatment and disease stage. Therefore, the observed differences in recurrence patterns should be considered exploratory and hypothesis-generating rather than definitive.

The strengths of this study include a large, single-institution cohort operated on by experienced gynecologic oncologists at a high-volume tertiary center, the availability of detailed clinicopathological data, and a long median follow-up period of 64.5 months. Furthermore, the analysis encompasses twenty years of data and includes a well-defined cohort, ensuring consistency in surgical and pathological evaluation. However, several limitations must be acknowledged. The retrospective design and lack of randomized design carry a risk of selection bias, as patients undergoing lymphadenectomy tend to present with higher-risk disease and are more frequently treated with adjuvant therapy. Higher pelvic lymph node counts were observed in patients with more advanced disease, indicating that lymph node count likely reflects surgical risk assessment and staging aggressiveness rather than an independent biological effect. Although multivariate analyses were performed, residual confounding cannot be excluded. Therefore, the absence of an independent association between pelvic lymph node count and survival outcomes should be interpreted within this clinical context. Because para-aortic lymphadenectomy was performed selectively based on clinical judgment, residual confounding related to surgical extent cannot be fully excluded. The absence of SLN use in the study also represents another limitation. An important limitation of this study is the absence of molecular tumor classification in accordance with the ESGO/ESTRO/ESP guidelines. In contemporary endometrial cancer management, molecular subgroups play a central role in prognostic assessment and adjuvant treatment decision-making. However, the primary objective of the present study was not to determine whether pelvic lymph node count represents an independent biological marker, but rather to evaluate the relationship between surgical extent and clinical outcomes. Accordingly, although the lack of molecular data limits the completeness of the dataset, it does not invalidate the principal interpretation that pelvic lymph node count reflects surgical staging intensity. Therefore, pelvic lymph node count should be interpreted as a parameter that more closely reflects the surgeon’s intraoperative risk assessment and staging approach than intrinsic tumor biology. In this context, any observed associations between lymph node count and oncologic outcomes should be regarded not as causal effects, but as reflections of underlying clinical decision-making processes.

## 5. Conclusions

In surgically staged endometrial cancer, a higher pelvic lymph node count (>20) was not independently associated with survival or recurrence outcomes after adjustment for established clinicopathologic factors. Survival was primarily determined by age, histologic subtype, FIGO stage, tumor grade, and lymphatic metastasis. Pelvic lymph node count appears to represent surgical staging intensity and intraoperative risk assessment rather than a definitive prognostic threshold. Accordingly, nodal extent should not be considered an isolated determinant of prognosis but rather interpreted within a risk-adapted clinical context. Although the absence of molecular classification limits the completeness of the dataset, it does not alter the study’s interpretative framework centered on surgical practice.

## Figures and Tables

**Table 1:** Comparison of clinical and pathological characteristics between pelvic lymph node count ≤20 and >20.

Characteristics	≤20 *(n* = 262)	>20 (*n* = 298)	*p* Value
Age, y, median (range)	63.0 (31.0–90.0)	63.0 (37.0–85.0)	0.323
BMI, kg/m^2^, median (range)	34.3 (22.0–65.3)	34.0 (18.0–61.5)	0.764
Ca 125, U/mL, median (range)	17.3 (5.0–1980.0)	19.5 (6.0–3250.0)	0.078
Tumor size, cm, median (range)	3.7 (0.2–10.5)	4.2 (0.4–15.3)	**0.002**
No. of PALN harvested, median (range)	11 (1.0–35.0)	18 (1.0–51.0)	**<0.001**
FIGO stage ^a^, *n,* (%) I II III	213 (81.3%) 13 (5.0%) 36 (13.7%)	206 (69.1%) 25 (8.4%) 67 (22.5%)	**<0.001**
Histologic subtype, *n,* (%) Endometrioid Non-endometrioid	214 (81.7%) 48 (18.3%)	242 (81.2%) 56 (18.8%)	0.973
Grade, *n*, (%) 1 2 3	103 (39.3%) 80 (30.5%) 79 (30.2%)	107 (35.9%) 96 (32.2%) 95 (31.9%)	0.457
Depth of myometrial invasion, *n,* (%) <50% ≥50%	189 (72.1%) 73 (27.9%)	193 (64.8%) 105 (35.2%)	0.075
LVSI, *n*, (%) Absent Present	188 (71.8%) 74 (28.2%)	212 (71.1%) 86 (28.9%)	0.947
Cervical stromal invasion, *n,* (%) Absent Present	236 (90.1%) 26 (9.9%)	255 (85.6%) 43 (14.4%)	0.136
Para-aortic Lymphadenectomy, *n*, (%) Yes No	183 (70.1%) 79 (29.9%)	253 (84.9%) 45 (15.1%)	**<0.001**
Lymphatic metastasis, *n*, (%) None Pelvic Para-aortic Pelvic + Para-aortic	229 (87.4%) 21 (8.0%) 4 (1.5%) 8 (3.1%)	245 (82.5%) 16 (5.1%) 9 (3.0%) 28 (9.4%)	**0.006**
Surgery type, *n*, (%) Laparoscopy Laparotomy	65 (24.8%) 197 (75.2%)	59 (19.8%) 239 (80.2%)	0.186
Adjuvant treatment, *n,* (%) None Chemotherapy Radiotherapy Chemotherapy and radiotherapy	61 (23.3%) 14 (5.3%) 129 (49.2%) 58 (22.1%)	54 (18.1%) 15 (5.0%) 140 (47.0%) 89 (29.9%)	**0.048**

Abbreviations: BMI: body mass index; FIGO: International Federation of Gynecology and Obstetrics; LVSI: lymph-vascular space invasion; PALN: Para-aortic Lymph Node, n: number; y: years. ^a^ The disease stage was based on the 2009 FIGO staging system.

**Table 2:** Univariate and multivariate analysis of factors affecting overall survival.

	Univariate Model		Multivariate Model	
	HR (95% CI)	*p*	HR (95% CI)	*p*
Age (per year)	1.087 (1.064–1.109)	**<0.001**	1.078 (1.055–1.103)	**<0.001**
BMI (per kg/m^2^)	1.001 (0.979–1.024)	0.903	-	-
Ca 125 (per unit)	1.004 (1.002–1.007)	**<0.001**	1.003 (1.000–1.005)	**0.034**
Tumor size (per cm)	1.110 (1.030–1.195)	**0.006**	1.066 (0.979–1.162)	0.140
No. of PALN harvested (per 1 node)	1.012 (0.987–1.022)	0.780	-	-
Pelvic LN ≤20 (Ref.) >20	1.00 0.932 (0.663–1.309)	0.683	-	-
Histologic subtype Endometrioid (Ref.) Non-Endometrioid	1.00 2.904 (2.025–4.177)	**<0.001**	2.343 (1.442–3.735)	**<0.001**
Para-aortic lymphadenectomy No (Ref.) Yes	1.00 1.032 (0.642–1.645)	0.915	-	-
Lymphatic metastasis Pelvic—None (Ref.) Para-aortic—None (Ref.) Pelvic+Para-aortic—None (Ref.)	1.152 (0.587–2.264) 1.861 (0.764–4.556) 3.404 (2.131–5.435)	0.685 0.174 **<0.001**	- - 3.745 (2.308–6.0851)	- - **<0.001**
FIGO Stage II—I (Ref.) III—I (Ref.)	0.632 (0.281–1.433) 2.254 (1.551–3.272)	0.271 **<0.001**	- 2.213 (1.36–3.60)	- **<0.001**
Depth of myometrial invasion <50% (Ref.) >50%	1.00 1.855 (1.320–2.608)	**<0.001**	1.292 (0.863–1.935)	0.213
LVSI Absent (Ref.) Present	1.00 1.687 (1.193–2.386)	**0.003**	0.939 (0.623–1.417)	0.766
Grade 2—1 (Ref.) 3—1 (Ref.)	0.893 (0.612–1.285) 2.221 (1.563–3.092)	0.524 **<0.001**	- 2.845 (1.734–4.673)	- **<0.001**
Surgical type Laparoscopy (Ref.) Laparotomy	1.00 1.269 (0.733–2.162)	0.409	-	-
Adjuvant Treatment CT—(Ref: None) RT—(Ref: None) CRT—(Ref: None)	1.178 (0.537–2.404) 0.836 (0.593–1.176) 1.598 (1.113–2.276)	0.660 0.290 **0.012**	- - 0.724 (0.365–0.824)	- - **0.039**

Abbreviations: HR: hazard ratio; CI: confidence interval; BMI: body mass index; FIGO: International Federation of Gynecology and Obstetrics; LVSI: lymph-vascular space invasion, LN: Lymph Node, PALN: Para-aortic Lymph Node, CT: Chemotherapy, RT: Radiotherapy, CRT: Chemotherapy and Radiotherapy, HR: Hazard Ratio, CI: Confidence Interval, Ref: Reference Category. Footnote: Variables included in the multivariate Cox regression model were selected based on clinical relevance and univariate analysis results. Adjuvant treatment was included in the final multivariate model to address potential treatment-related confounding. Reference categories (Ref.) are indicated for all categorical variables.

**Table 3:** Univariate and multivariate analysis of factors affecting disease-free survival.

	Univariate Model		Multivariate Model	
	HR (95% CI)	*p*	HR (95% CI)	*p*
Age (per year)	1.042 (1.014–1.071)	**0.003**	1.032 (1.002–1.063)	**0.034**
BMI (per kg/m^2^)	1.004 (0.973–1.036)	0.813	-	-
Ca 125 (per unit)	1.002 (0.998–1.006)	0.350	-	-
Tumor size (per cm)	1.112 (1.006–1.229)	**0.037**	1.044 (0.932–1.170)	0.457
No. of PALN harvested (per 1 node)	1.132 (0.973–1.025)	0.758	-	-
Pelvic LN ≤20 (Ref.) >20	1.00 1.016 (0.629–1.641)	0.949	-	-
Histologic subtype Endometrioid (Ref.) Non-Endometrioid	1.00 6.496 (4.004–10.525)	**<0.001**	3.906 (2.004–7.616)	**<0.001**
Para-aortic lymphadenectomy No (Ref.) Yes	1.00 1.534 (0.764–3.092)	0.235	-	-
Lymphatic metastasis Pelvic—None (Ref.) Para-aortic—None (Ref.) Pelvic + Para-aortic—None (Ref.)	1.405 (0.563–3.494) 2.176 (0.682–6.923) 4.894 (2.751–8.704)	0.468 0.189 **<0.001**	- - 5.567 (3.013–10.285)	- - **<0.001**
FIGO Stage II—I (Ref.) III—I (Ref.)	0.889 (0.325–2.416) 3.643 (2.234–5.947)	0.802 **<0.001**	- 2.860 (1.535–5.342)	- **<0.001**
Depth of myometrial invasion <50% (Ref.) ≥50%	1.00 1.887 (1.166–3.054)	**0.010**	1.097 (0.611–1.969)	0.756
LVSI Absent (Ref.) Present	1.00 2.274 (1.407–3.676)	**<0.001**	1.121 (0.631–1.991)	0.697
Grade 2—1 (Ref.) 3—1 (Ref.)	0.476 (0.268–0.866) 4.611 (2.807–7.608)	**0.015** **<0.001**	1.069 (0.473–2.395) 0.983 (0.341–2.784)	0.890 **<0.001**
Surgical type Laparoscopy (Ref.) Laparotomy	1.00 1.187 (0.600–2.332)	0.623	-	-
Adjuvant Treatment CT—(Ref: None) RT—(Ref: None) CRT—(Ref: None)	2.353 (1.084–5.185) 0.604 (0.372–0.995) 2.758 (1.706–4.457)	**0.031** **0.044** **<0.001**	1.124 (0.623–2.011) 0.891 (0.542–1.461) 1.282 (0.781–2.103)	0.714 0.678 0.322

Abbreviations: HR: hazard ratio; CI: confidence interval; BMI: body mass index; FIGO: International Federation of Gynecology and Obstetrics; LVSI: lymph-vascular space invasion, LN: Lymph Node, PALN: Para-aortic Lymph Node, CT: Chemotherapy, RT: Radiotherapy, CRT: Chemotherapy and Radiotherapy, HR: Hazard Ratio, CI: Confidence Interval, Ref: Reference Category. Footnote: Variables included in the multivariate Cox regression model were selected based on clinical relevance and univariate analysis results. Adjuvant treatment was included in the final multivariate model to address potential treatment-related confounding. Reference categories (Ref.) are indicated for all categorical variables.

**Table 4:** Recurrence outcomes according to the number of pelvic lymph nodes harvested (≤20 vs. >20).

Characteristics	≤20 (n = 262)	>20 (n = 298)	*p* Value
Recurrence, n, (%) Yes No	32 (12.2%) 230 (87.8%)	35 (11.7%) 263 (88.3%)	0.968
Recurrence site Local Distant Local + Distant	n = 32 6 (18.8%) 21 (65.6%) 5 (15.6%)	n = 35 18 (51.4%) 13 (37.1%) 4 (11.4%)	**0.019**

## Data Availability

The original contributions presented in this study are included in the article. Further inquiries can be directed to the corresponding authors.

## Supplementary Materials

The following supporting information can be downloaded at: https://www.mdpi.com/article/10.3390/medicina62020399/s1, Figure S1: Heatmap of survival-related differences across pelvic lymph node thresholds; Table S1a: Exploratory sensitivity analysis of pelvic lymph node cut-offs for overall survival; Table S1b: Exploratory sensitivity analysis of pelvic lymph node cut-offs for disease-free survival.

## Abbreviations

The following abbreviations are used in this manuscript:

BMI	Body Mass Index
Ca 125	Cancer antigen 125
DFS	Disease-free survival
ESGO	European Society of Gynaecological Oncology
ESTRO	European Society for Radiotherapy and Oncology
ESP	European Society of Pathology
FIGO	International Federation of Obstetrics and Gynecology
LVSI	Lymphovascular space invasion
LN	Lymph node
OS	Overall survival
PALN	Para-aortic Lymph Node
POLE	DNA polymerase epsilon exonuclease domain
PLN	Pelvic lymph node
SEER	Surveillance, Epidemiology and Outcomes
SLN	Sentinel lymph node
