# Supplementary material for: Prognostic Impact of Pelvic Lymph Node Count in Surgically Staged Endometrial Cancer
Source: Medicina (Kaunas). 2026 Feb 19;62(2):399. doi: 10.3390/medicina62020399 (PMC12942316; doi:10.3390/medicina62020399)
Supplement: Supplementary file 1 [file medicina-62-00399-s001.zip › medicina-4140553-supplementary.pdf]

Supplementary Table S1a. Exploratory sensitivity analysis of pelvic lymph node cut-offs for overall survival

Cut-off	N ≤ cut-off	Deaths ≤ cut-off	Death rate ≤ cut-off	N > cut-off	Deaths > cut-off	Death rate > cut-off	Death rate difference
5.0	16.0	4.0	0.25	544.0	130.0	0.239	0.011
6.0	21.0	4.0	0.19	539.0	130.0	0.241	-0.051
7.0	27.0	6.0	0.222	533.0	128.0	0.24	-0.018
8.0	32.0	9.0	0.281	528.0	125.0	0.237	0.045
9.0	39.0	12.0	0.308	521.0	122.0	0.234	0.074
10.0	50.0	14.0	0.28	510.0	120.0	0.235	0.045
11.0	62.0	18.0	0.29	498.0	116.0	0.233	0.057
12.0	80.0	25.0	0.312	480.0	109.0	0.227	0.085
13.0	99.0	28.0	0.283	461.0	106.0	0.23	0.053
14.0	120.0	31.0	0.258	440.0	103.0	0.234	0.024
15.0	143.0	39.0	0.273	417.0	95.0	0.228	0.045
16.0	166.0	46.0	0.277	394.0	88.0	0.223	0.054
17.0	180.0	50.0	0.278	380.0	84.0	0.221	0.057
18.0	209.0	57.0	0.273	351.0	77.0	0.219	0.053
19.0	232.0	64.0	0.276	328.0	70.0	0.213	0.062
20.0	262.0	69.0	0.263	298.0	65.0	0.218	0.045
21.0	284.0	73.0	0.257	276.0	61.0	0.221	0.036
22.0	304.0	78.0	0.257	256.0	56.0	0.219	0.038
23.0	337.0	87.0	0.258	223.0	47.0	0.211	0.047
24.0	357.0	95.0	0.266	203.0	39.0	0.192	0.074
25.0	378.0	102.0	0.27	182.0	32.0	0.176	0.094
26.0	395.0	105.0	0.266	165.0	29.0	0.176	0.09
27.0	415.0	110.0	0.265	145.0	24.0	0.166	0.1
28.0	427.0	111.0	0.26	133.0	23.0	0.173	0.087
29.0	441.0	113.0	0.256	119.0	21.0	0.176	0.08
30.0	455.0	115.0	0.253	105.0	19.0	0.181	0.072
31.0	466.0	117.0	0.251	94.0	17.0	0.181	0.07
32.0	478.0	119.0	0.249	82.0	15.0	0.183	0.066
33.0	485.0	121.0	0.249	75.0	13.0	0.173	0.076
34.0	493.0	123.0	0.249	67.0	11.0	0.164	0.085
35.0	499.0	124.0	0.248	61.0	10.0	0.164	0.085
36.0	507.0	125.0	0.247	53.0	9.0	0.17	0.077
37.0	512.0	127.0	0.248	48.0	7.0	0.146	0.102
38.0	518.0	129.0	0.249	42.0	5.0	0.119	0.13
39.0	520.0	129.0	0.248	40.0	5.0	0.125	0.123
40.0	528.0	131.0	0.248	32.0	3.0	0.094	0.154

Supplementary Table S1b. Exploratory sensitivity analysis of pelvic lymph node cut-offs for disease-free survival

Cut-off	N ≤ cut-off	Recurrences ≤ cut-off	Recurrence rate ≤ cut-off	N > cut-off	Recurrences > cut-off	Recurrence rate > cut-off
5.0	16.0	1.0	0.062	544.0	66.0	0.121
6.0	21.0	1.0	0.048	539.0	66.0	0.122
7.0	27.0	3.0	0.111	533.0	64.0	0.12
8.0	32.0	5.0	0.156	528.0	62.0	0.117
9.0	39.0	5.0	0.128	521.0	62.0	0.119

10.0	50.0	7.0	0.14	510.0	60.0	0.118
11.0	62.0	8.0	0.129	498.0	59.0	0.118
12.0	80.0	9.0	0.112	480.0	58.0	0.121
13.0	99.0	12.0	0.121	461.0	55.0	0.119
14.0	120.0	14.0	0.117	440.0	53.0	0.12
15.0	143.0	19.0	0.133	417.0	48.0	0.115
16.0	166.0	25.0	0.151	394.0	42.0	0.107
17.0	180.0	26.0	0.144	380.0	41.0	0.108
18.0	209.0	29.0	0.139	351.0	38.0	0.108
19.0	232.0	30.0	0.129	328.0	37.0	0.113
20.0	262.0	32.0	0.122	298.0	35.0	0.117
21.0	284.0	34.0	0.12	276.0	33.0	0.12
22.0	304.0	35.0	0.115	256.0	32.0	0.125
23.0	337.0	41.0	0.122	223.0	26.0	0.117
24.0	357.0	46.0	0.129	203.0	21.0	0.103
25.0	378.0	50.0	0.132	182.0	17.0	0.093
26.0	395.0	52.0	0.132	165.0	15.0	0.091
27.0	415.0	53.0	0.128	145.0	14.0	0.097
28.0	427.0	55.0	0.129	133.0	12.0	0.09
29.0	441.0	57.0	0.129	119.0	10.0	0.084
30.0	455.0	58.0	0.127	105.0	9.0	0.086
31.0	466.0	59.0	0.127	94.0	8.0	0.085
32.0	478.0	60.0	0.126	82.0	7.0	0.085
33.0	485.0	60.0	0.124	75.0	7.0	0.093
34.0	493.0	60.0	0.122	67.0	7.0	0.104
35.0	499.0	60.0	0.12	61.0	7.0	0.115
36.0	507.0	61.0	0.12	53.0	6.0	0.113
37.0	512.0	62.0	0.121	48.0	5.0	0.104
38.0	518.0	64.0	0.124	42.0	3.0	0.071
39.0	520.0	64.0	0.123	40.0	3.0	0.075
40.0	528.0	65.0	0.123	32.0	2.0	0.062

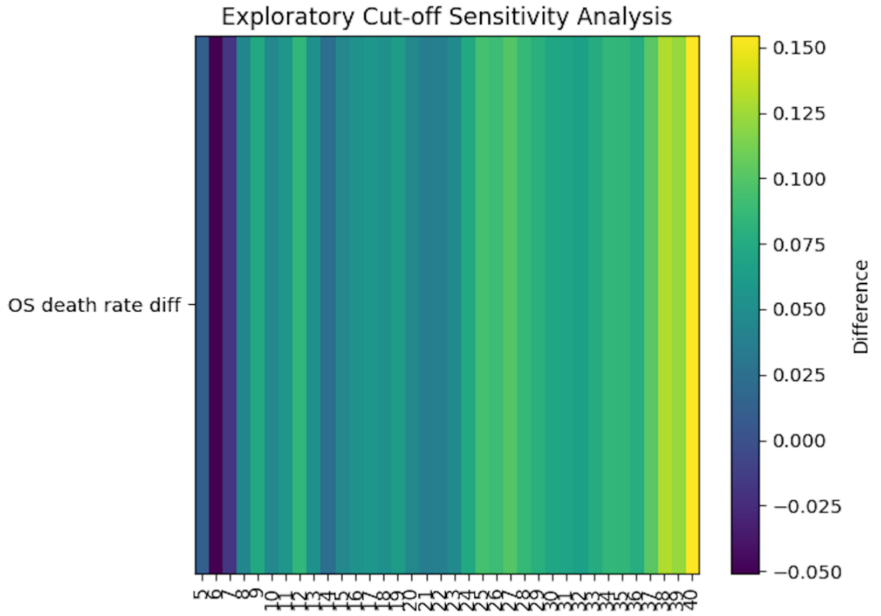

Supplementary Figure S1. Heatmap of survival-related differences across pelvic lymph node thresholds
